# Digital mental health care: five lessons from Act 1 and a preview of Acts 2–5

**DOI:** 10.1038/s41746-023-00760-8

**Published:** 2023-01-26

**Authors:** Thomas Insel

**Affiliations:** Exec Chair, Vanna Health; Adj Prof, Dept of Psychiatry and Behavioral Science, Stanford University School of Medicine, Stanford, CA USA

**Keywords:** Health care, Health services, Health policy, Psychology

## Abstract

Digital tools are transforming mental health care. The promise of this transformation to improve outcomes has not yet been realized fully. While some have become skeptical, this article argues that we are just at the end of Act 1, with several opportunities and challenges ahead.

The past decade has witnessed the birth of a new industry, sometimes labeled the digital mental health revolution^1^. Already there have been extraordinary developments: over $10B invested by venture capital firms since 2017, 10 new companies valued at over $1B, and hundreds of thousands of patients receiving care from telepsychiatry companies that did not exist five years ago (https://rockhealth.com/insights/2021-year-end-digital-health-funding-seismic-shifts-beneath-the-surface/). Collectively, these investments and innovations represent a significant change but I believe only the beginning, arguably Act 1, of a transformation of mental health care.

Perhaps signaling the end of this first Act, the fever of excitement in digital mental health innovation recently appears to be cooling. One of the fastest growing companies is being investigated by the Department of Justice for alleged over-prescribing of psychiatric medications (https://www.fiercehealthcare.com/health-tech/cerebral-under-federal-investigation-possible-violation-controlled-substances-law). A Senate investigation is looking at potential privacy violations from three of the largest tele-mental health companies (https://www.warren.senate.gov/imo/media/doc/2022.06.22%20Letter%20to%20Mental%20Health%20Apps%20on%20Data%20Privacy%20and%20Sharing1.pdf). Some of the new generation of companies that have gone public have not fared well economically (https://bhbusiness.com/2022/11/08/talkspace-names-new-ceo-reveals-layoffs-as-part-of-turnaround-effort/). And academic research has reported depressingly low levels of engagement with digital mental health products, lack of integration with electronic medical records, and little evidence of efficacy from randomized clinical trials^2^.

To the extent that the digital mental health revolution was hyped as a solution to the mental health crisis, this reassessment is both timely and inevitable. But the critics of digital mental health technology should recognize that this field is still young. How it develops over the next ten years depends on learning the lessons of this first Act, including a full reckoning with mistakes and a full accounting of the challenges ahead. If this is the end of Act 1, perhaps this is an opportune moment to consider what Acts 2 through 5 will offer. Here I suggest five lessons to take from this early phase of the digital mental health revolution. And then I share a potential narrative for the subsequent acts if technology is to transform outcomes for people with mental illness.

## Lessons from Act 1

1. Technology is only part of the answer.
  Many of the most successful companies in this first act have basically digitized existing services, making them more widely available. Whether for diagnostics or therapeutics, the early promise that artificial intelligence and autonomous agents would replace human intervention has not been realized. In spite of the power of AI (artificial intelligence) for deriving insights from medical records^3^ or the sensitivity of digital phenotyping^4^, diagnosis still involves a human interviewer collecting patient data. Similarly, while companies creating autonomous agents and digital therapeutics have been funded robustly, most studies show that psychotherapy requires a human in the loop to optimize engagement^5^. Nevertheless, there is a continuing opportunity for objective data to augment subjective data through digital measures of sleep or activity or sociality^6^. And natural language processing should be able to add measures of sentiment and semantic coherence to a clinician’s assessment from a diagnostic interview^7^. Technology will undoubtedly play an increasing role in psychiatry – inevitably replacing much of what human providers do today - but thus far technology has been primarily a tool for scaling, training providers, and improving clinical care and not a replacement for clinical experts.
2. Digital mental health companies are not traditional tech companies.
  More than any piece of software or hardware, the culture of tech companies may have the greatest short-term influence on mental health care. Tech companies focus on user experience, data collection, and iterative learning – all attributes that can improve the impact of mental health care. But other aspects of venture-backed tech culture, such as the “move fast and break things” approach that stresses disruption, rapid growth, and rapid returns to investors have not been helpful. Mental health companies founded by software engineers or computer scientists without a deep understanding of clinical complexity may find themselves building products without users or solving for the wrong problems. This problem, known in start-ups as the product-market fit problem, can be addressed by an effective Chief Medical Officer, but that role has been undervalued in many digital mental health companies. Very few behavioral health companies have been led by behavioral health experts.
3. The business model for health care does not always work for digital mental health.
  In contrast to other areas of medicine, much of psychiatric care is paid for out of pocket and fewer providers accept insurance^8^. While a direct-to-consumer approach might seem to make sense for online mental health care, this approach has proven expensive and difficult for digital mental health companies. Expensive because online marketing has become competitive, with the cost of ads and optimization requiring start-ups to raise huge investments to acquire online “users”. And difficult because companies suffer from negative online reviews when they don’t give patients what they want, an inevitable consequence of treating patients with addictions or self-destructive behaviors. Not surprising therefore, that many companies that began as direct to consumer providers evolved to contract with employers or health plans. Some companies have seen low adoption of their therapeutic apps when included as a benefit for employees and most pilots with healthcare payers have been slow to launch and failed to scale for widespread implementation. Although digital technologies may work well in a value-based health care system, most behavioral health care is reimbursed by fee for service that may undervalue care provided by video, text, or chat and may not reward digital therapeutics.
4. There is a gap between innovation and public health impact.
  Little of the venture-capital investment in behavioral health start*-*ups has gone to the most severely ill patients^9^. The need for innovation is no less for young people with psychosis or patients with complex co-morbid conditions, but entrepreneurs have generally avoided the “deep end of the pool” in favor of patients with mild to moderate behavioral health issues. In one sense this is surprising, since people with serious mental illness and co-morbid medical conditions are among the most expensive in our health care system. But the simplicity and broad appeal of a mindfulness app or online cognitive-behavior therapy for mild anxiety and depression have trumped the public health needs of more complex psychiatric states.
5. The absence of regulation hurts developers, patients, and providers.

While regulation is often considered a barrier to innovation, the mental health innovation community has been challenged by the absence of a regulatory framework. In contrast to drug development for which the FDA has clear guidelines for efficacy and safety, there is no agency that regulates either face-to-face or remote psychotherapy. The FDA has attempted to review software as a medical device, but the continually iterating world of software development does not conform to the FDA process, nor does it lend itself to traditional randomized clinical trials. Moreover, there is no clear process for monitoring adverse events, analogous to the surveillance system used for medications. Without a process for defining efficacy and safety, both the public and the provider community have found it difficult to navigate the world of apps and online therapies.

## Quo Vadis

What’s next? This first Act has largely focused on solving problems of access by connecting patients and providers via telehealth. We have already met some of the main characters: companies valued at over $1 billion dollars connecting hundreds of thousands of patients to tens of thousands of providers. Those who rue the unresolved digital divide might note that technology has already begun to democratize care relative to the previous brick and mortar system.

Act 1 will end with millions of people receiving mental health care for the first time. But access alone will not improve outcomes and, to be clear, telehealth may be the most financially successful but it is the least innovative part of the digital mental health revolution. There is much more to be done in what I describe as the subsequent acts, recognizing that, in contrast to a play, much of this work will advance simultaneously.

Act 2 will need to focus on quality of care with resolution of privacy issues, integration with primary care, improved training of the provider workforce, and demonstration of improved outcomes. The integration of digital mental health tools with telehealth to create telehealth2.0 can be a near-term step. In Act 1, we saw the pressure from investors to scale quickly and push efficiency (more patients per provider), sometimes sacrificing quality for quantity. Act 2 can improve quality by prioritizing value (clinical outcomes) over volume. Innovative payment models, such as value-based contracts, will be helpful to incentivize adoption of tools that improve outcomes. In Act 2, we will likely see the horizontal integration of services, moving from point solutions to comprehensive platforms for stepped care.

Act 3 will hopefully focus on equity and engagement, ensuring that those who have not been engaged with our care system, either because they are from a marginalized group or are distrustful of care, will have access to high quality care. Innovations that focus upstream of crisis care promise to mitigate both the high expense and the dire outcomes of serious mental illness.

In Act 4 digital mental health will become a mainstream of behavioral health care as part of public and private insurance, guided by a regulatory process that ensures efficacy, effectiveness, and safety. That regulatory process will require a new public-private partnership, different from the process currently used for drug development, with an eye to real world evidence as well as more traditional clinical trials.

Finally, Act 5 will provide population impact with reductions in morbidity and mortality from mental illness. In spite of more people receiving treatment and more money spent on mental health care, population outcomes have deteriorated over the past decade^10^. The ultimate success and global promise of the digital mental health revolution will be its impact on life expectancy and well-being for those with mental illness.

One risk to remember in predicting the next phase of innovation for mental health is that technology is not only changing rapidly, it’s progressing on a non-linear course. As an example, the use of AI to predict 3-dimensional protein structure was reported in July, 2021^11^. The use of the same algorithm to predict over 200 million protein structures was reported in 2022 (https://alphafold.ebi.ac.uk/). AI tools for analyzing speech, voice, and face emotion as well as tools for generating conversational language are evolving at a rate that surpasses traditional biomedical or cognitive research. These tools may change the fundamentals of how we diagnose and treat mental illness, not only by upskilling providers but by creating a new class of autonomous providers that are more engaging than what we have seen thus far.

But whatever the path of innovation, successful tech companies may transform mental health care not only with software and hardware, but with a culture that is fundamentally different from the traditional provider-payer approach and more suited to the preferences of the generation that has grown up expecting to receive goods and services with convenience and choice. Successful tech companies build for their “users”, designing products based on deep research about user preferences and needs. It seems likely, although as yet unproven, that this kind of user-based design will prove helpful for increasing engagement in mental health care, especially for a young generation of digital natives. Successful tech companies adopt a data-driven approach, collecting outcomes continually to know how to improve engagement and retention. This is, of course, the basis for the addictive force of social media. But the lesson for mental health care, which notoriously has not been able to engage those with serious mental illness and has failed to adopt measurement-based care, is that data can improve outcomes. And finally, successful tech companies iterate or adapt based on the data collected. Few traditional mental health care systems have shown this kind of rapid ability to pivot based on outcomes or opportunities. Digital tools can be the path for creating a learning-based mental health care system.

Finally, a call to action. As we take stock of this first phase of the digital mental health revolution, we should recognize that these are early days in a journey that will likely take at least another decade to show population impact. Rather than focusing on the failure of the revolution, now is the time to learn from Act 1 and begin the critical work of the subsequent acts. The tools are available, the pathway is clear, and with the growing mental health crisis, the need is urgent. We need a massive effort, both public and private, to move from access (Act 1) to the next steps of engagement, integration, quality improvement, and better outcomes. Investors and payers now need to focus more on outcomes as much as access, innovating on payment as well as tech solutions. As public insurance (Medicaid and Medicare) is the largest payer of care for people with serious mental illness, the federal government could use the Certified Community Behavioral Health Centers to develop a value-based approach for improving equity and engagement using both high tech and high touch for the population with greatest mental health needs. A public-private effort, working with the FDA and NIH, could create the regulatory framework for digital mental health, including guidelines for quality, privacy, trust, and safety. We have seen remarkable public health successes when we identify clear goals and timelines. Digital mental health care can be the driver for such a success if we focus on the needs of people with mental illness and the steps required for recovery.

### Reporting summary

Further information on research design is available in the Nature Research Reporting Summary linked to this article.

## Supplementary information

Reporting Summary

Editorial Policy

## Data Availability

This commentary provides neither original nor novel data. All data herein are attributed to referenced publications.
